# WORKPLACE PERSPECTIVES ON CAREGIVER-FRIENDLY WORKPLACE POLICIES

**DOI:** 10.1093/geroni/igae098.1832

**Published:** 2024-12-31

**Authors:** Maryssa Pallis, Pamela Nadash

**Affiliations:** University of Massachusetts Boston, Boston, Massachusetts, United States; University of Massachusetts Boston, Boston, Massachusetts, United States

## Abstract

Roughly 60% of all family caregivers are employed, and it is well-established that they experience stresses related to work-life balance, which may cause them to leave employment altogether. Thus, an important goal of the 2022 National Strategy to Support Family Caregivers focuses on caregivers’ financial security, specifically noting the importance of workplace protections and supports. This presentation provides a workplace perspective on caregiver-friendly workplace policies, drawing on data collected to support the RAISE Family Caregiving Advisory Council, created under the Recognize, Assist, Include, Support, and Engage (RAISE) Family Caregivers Act (2018). It draws on a data collection effort involving listening sessions, focus groups, and key informant interviews with over 150 entities (including employers and employer intermediaries). The study aimed to identify what employers had to say about the challenges and benefits of creating caregiver-friendly workplaces. Issues raised included the importance of workplace policies such as paid family and medical leave, flexible work policies, and caregiver support programs. Findings suggest consensus among employers regarding the need for caregiver-friendly workplace policies; however, barriers to creating a supportive culture included caregivers’ reluctance to self-identify and utilize formal policies. Beyond adopting formal caregiver-friendly workplace polices, the most critical area for action would be to explore best practices for proactively integrating a caregiver-friendly workplace culture alongside formal policy interventions.

